# Highly active and durable WO_3_/Al_2_O_3_ catalysts for gas-phase dehydration of polyols†

**DOI:** 10.1039/d0ra08340b

**Published:** 2020-10-12

**Authors:** Takeshi Aihara, Katsuya Asazuma, Hiroki Miura, Tetsuya Shishido

**Affiliations:** Department of Applied Chemistry for Environment, Graduate School of Urban Environmental Sciences, Tokyo Metropolitan University 1-1 Minami-Osawa Hachioji Tokyo 192-0397 Japan; Research Center for Hydrogen Energy-based Society, Tokyo Metropolitan University 1-1 Minami-Osawa Hachioji Tokyo 192-0397 Japan; Research Center for Gold Chemistry, Tokyo Metropolitan University 1-1 Minami-Osawa Hachioji Tokyo 192-0397 Japan; Elements Strategy Initiative for Catalysts & Batteries, Kyoto University Katsura, Nishikyo-ku Kyoto 615-8520 Japan

## Abstract

Gas-phase glycerol dehydration over WO_3_/Al_2_O_3_ catalysts was investigated. WO_3_ loading on γ-Al_2_O_3_ significantly affected the yield of acrolein and the catalyst with 20 wt% WO_3_ loading showed the highest activity. The WO_3_/Al_2_O_3_ catalyst with 20 wt% WO_3_ loading showed higher activity and durability than the other supported WO_3_ catalysts and zeolites. The number of Brønsted acid sites and mesopores of the WO_3_/Al_2_O_3_ catalyst did not decrease after the reaction, suggesting that glycerol has continuous access to Brønsted acid sites inside the mesopores of WO_3_/Al_2_O_3_, thereby sustaining a high rate of formation of acrolein. Dehydration under O_2_ flow further increased the durability of the WO_3_/Al_2_O_3_ catalyst, enabling the sustainable formation of acrolein. In addition, the WO_3_/Al_2_O_3_ catalyst with 20 wt% WO_3_ loading showed high activity for the dehydration of various polyols to afford the corresponding products in high yield.

## Abstract 2

Gas-phase glycerol dehydration over WO_3_/Al_2_O_3_ catalysts was investigated.

## Graphical abstract

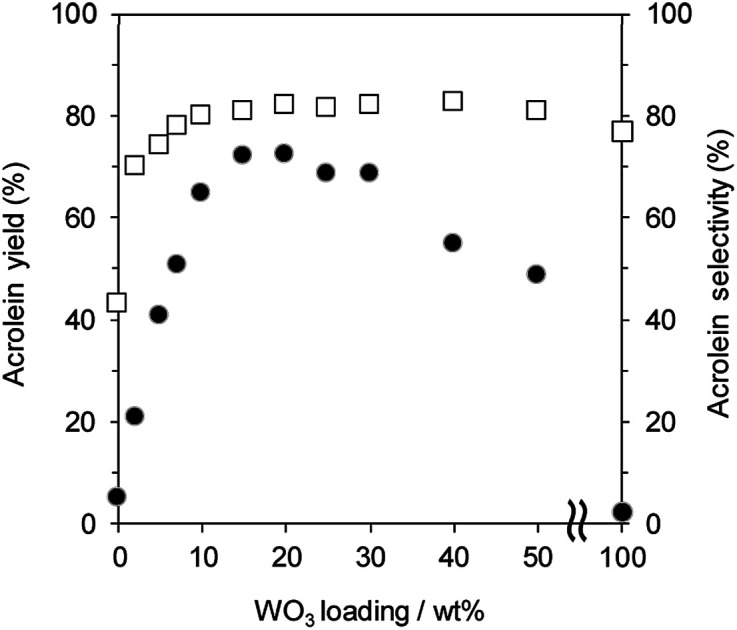

## Introduction

The conversion of biomass-derived compounds instead of oil-derived resources to valuable chemicals is essential for the sustainable development of society.^1–5^ Glycerol is one of the most important biomass-derived compounds. It is produced by the hydrolysis of glycerides such as those in vegetable oils and animal fats.^6–8^ A wide range of commodity chemicals can be produced from glycerol through various reactions such as dehydration,^9–25^ hydrogenolysis^26,27^ and oxidation^28–30^ over metal oxides, zeolites, metal phosphides and metal–organic frameworks. The dehydration of glycerol is a valuable reaction for the formation of acrolein, which is an important intermediate for the production of valuable compounds in the chemical and agricultural industries, such as acrylic acid and dl-methionine.^31–34^ Since acrolein is produced by the oxidation of propylene over a Bi/Mo-mixed oxide catalyst,^35–40^ a method for the production of acrolein from glycerol instead of oil-derived resources should be quite valuable.

Furthermore, solid acid-catalyzed dehydration of polyols has been reported to be a useful method for obtaining value-added chemicals (*e.g.* diols, aldehydes, ketones and alkenes).^41–48^ Many of these reactions, however, usually require a low substrate feed rate and high reaction temperatures to achieve a high conversion rate. Hence, the development of a catalyst that shows high activity and selectivity to give the target products under mild conditions is still a challenge.

On the other hand, the deposition of coke on the catalyst surface contributes to deactivation of the catalyst in dehydration. The mechanism of coke formation and the relationship between coke formation and acid strength and/or porosity of the catalyst have been widely investigated.^49–54^ Two deactivation mechanisms due to coke formation have been proposed so far; the direct deposition of coke at catalytically active acid sites and coke formation at the entrance of micropores, which hinders the substrate from accessing the active site inside the pore. A better understanding of the deactivation mechanism could be useful for the design of novel dehydration catalysts with high durability.

In the course of our investigation of the relationship between the structure of a two-dimensional tungsten oxide monolayer supported on metal oxides and their performance as acid catalysts,^55,56^ we reported that supported WO_3_ catalysts showed high activity for selective biomass conversion, such as the hydrogenolysis of glycerol and tetrahydrofurfuryl alcohol.^57–60^ These results encouraged us to further explore the application of selective conversion of biomass-derived chemicals over supported WO_3_ catalysts, in particular polyols to give value-added chemicals.

In this study, gas-phase glycerol dehydration over WO_3_/Al_2_O_3_ catalysts was investigated. The optimization of WO_3_ loading on Al_2_O_3_ enabled us to devise highly active and durable catalysts for glycerol dehydration. To reveal the effect of coke deposition at the catalyst surface on catalytic activity, the reaction under O_2_ flow and a detailed characterization were carried out. Furthermore, the application of WO_3_/Al_2_O_3_ catalyst to the dehydration of various polyols was investigated.

## Experimental

### Materials

Glycerol was purchased from Nacalai Tesque, Japan. Polyols, including ethylene glycol, 1,2-propanediol, 1,2-butanediol, 1,3-butanediol, 1,4-butanediol and 1,2-pentanediol, were purchased from Wako Pure Chemical Industries and Tokyo Chemical Industry, Japan. γ-Al_2_O_3_ (JRC-ALO-8), ZrO_2_(JRC-ZRO-3, monoclinic), TiO_2_(JRC-TIO-4, rutile) and Nb_2_O_5_ (JRC-NBO-1, orthorhombic) were supplied by Japan Reference Catalyst. (NH_4_)_10_W_12_O_41_·5H_2_O and WO_3_ were purchased from Wako Pure Chemical Industries, Japan. All zeolites such as H-ZSM-5(90), H-β(25), H-Y(5.5) and H-MOR(20), namely, JRC-Z5-90H, JRC-Z-HB25, JRC-Z-HY5.5 and JRC-Z-HM20, where the number in parentheses is the SiO_2_/Al_2_O_3_ ratio, were supplied by Japan Reference Catalyst.

### Catalyst preparation

Metal oxides and zeolites were calcined at 773 K for 3 h in flowing air. A series of WO_3_-loaded catalysts were prepared by impregnation of supports with an aqueous solution of (NH_4_)_10_W_12_O_42_, dried at 353 K for 6 h, and then calcined at 1123 K for 3 h in flowing air.^55,56^ The surface coverages of Al_2_O_3_, ZrO_2_ and TiO_2_ with a WO_3_ monolayer were estimated by using the cross-sectional area of a WO_6_ octahedral unit (0.22 nm^−2^)^61^ and were almost 100% at 20, 10 and 2.5 wt% WO_3_ loading, respectively.^60^

### Catalytic dehydration of polyols

Catalytic polyol dehydration was performed in a fixed-bed down-flow glass reactor with an inner diameter of 6 mm at an ambient pressure of N_2_ and O_2_. Prior to the reaction, 100 mg of a catalyst was placed in the catalyst bed, and the catalyst was treated at 588 K with N_2_ for 1.5 h. A 10 mol% aqueous solution of polyols was fed through the top of the reactor at a prescribed liquid feed rate together with a carrier gas flow of 20 mL min^−1^. The total substrate feed rate was 5.1 mmol h^−1^ and the composition of the mixture gases was substrate/H_2_O/carrier gas = 1/9/10 (molar ratio). The liquid products were collected in an ice trap (273 K) and a dry ice–methanol trap (195 K) every hour, and analysed by a FID-GC (GC2014, Shimadzu) with a 30 m capillary column of Stabilwax (GL Science, Japan). Gaseous products were analysed by on-line TCD-GC (GC-8A, Shimadzu, Japan) with a 3 m packed column (Porapak-Q, GL Science, Japan). For all the catalysts tested, the carbon balance was >90%, with a few exceptions.

### Characterization

X-ray diffraction (XRD) patterns of the catalyst were recorded by Rigaku SmartLab with Cu Kα radiation. The samples were scanned from 2*θ* = 10–70° at 10° min^−1^ and a resolution of 0.01°. X-ray photoelectron spectroscopy (XPS) analysis of the catalysts was performed using a JEOL JPS-9010 MX instrument. The spectra were measured using Mg Kα radiation. All spectra were calibrated using C 1s (284.5 eV) as a reference. The surface properties of samples were obtained from N_2_ isotherms obtained using a BELSORP-mini II (MicrotracBEL, Japan) at 77 K. The analysed samples were evacuated at 573 K for 3 h prior to the measurement. The surface area was estimated by the Brunauer–Emmett–Teller (BET) method. Micropore size distributions were determined by *t*-plot and micropore (MP) analysis. The Barrett–Joyner–Halenda (BJH) method was used to determine mesopore size distributions. FT-IR spectra were recorded by FT/IR-4200 typeA (JASCO, Japan) with a resolution of 4 cm^−1^. A total of 64 scans were averaged for each spectrum. Each sample (30 mg) was pressed into a self-supporting wafer with a diameter of 20 mm. Catalysts were pretreated under 20 kPa of flowing O_2_ at 773 K for 1.5 h and then evacuated. Catalysts were exposed to pyridine (0.5 kPa) at 303 K for 30 min, and then evacuated at 423 K for 30 min. NH_3_ temperature programmed desorption (NH_3_-TPD) was measured using a BELCAT II (MicrotracBEL, Japan). Fifty mg of the sample was loaded in a quartz reactor and pretreated under a He flow at 773 K for 1 h. NH_3_ adsorption was carried out over 30 min with 5% NH_3_/He at 373 K followed by purging with He for 15 min. The temperature was linearly increased from 373–873 K (10 K min^−1^). The outlet flow was analysed by means of a TCD and Q-Mass (BELMASS, MicrotracBEL, Japan). Thermogravimetric analyses (TG) were performed using a DTG-60H (Shimadzu, Japan) The temperature range was 303–1273 K (10 K min^−1^).

## Results and discussion

**Fig. 1:**
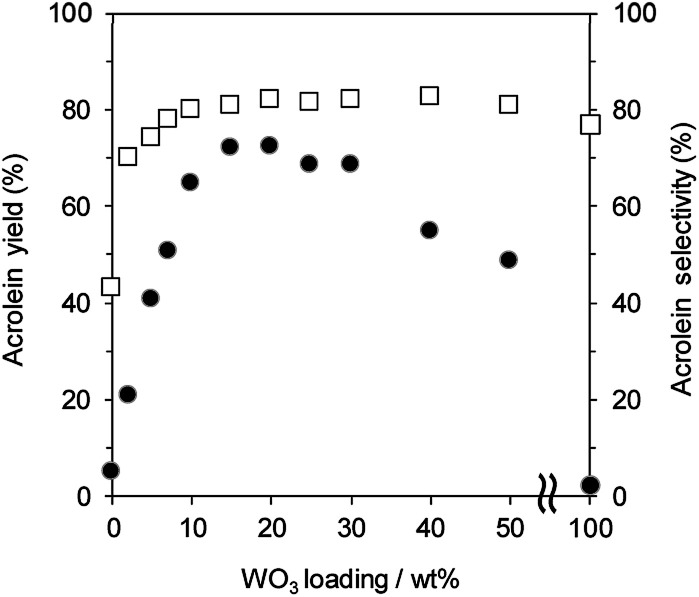
Dehydration of glycerol to acrolein over WO_3_/Al_2_O_3_ catalysts with various WO_3_ loadings. ●: yield, □: selectivity. Conditions: catalyst (100 mg), WHSV by glycerol (4.7 h^−1^), *T* = 588 K.

Fig. 1 shows the effect of WO_3_ loading over γ-Al_2_O_3_ on the gas-phase dehydration of glycerol at 588 K with WHSV by glycerol of 4.7 h^−1^. The catalysts with 0 and 100 wt% WO_3_ loading were γ-Al_2_O_3_ and bulk WO_3_, respectively. Dehydration over WO_3_/Al_2_O_3_ provided acrolein as a main product together with the formation of hydroxyacetone as a by-product. The selectivity for acrolein was around 80% regardless of the WO_3_ loading on γ-Al_2_O_3_. In contrast, the WO_3_ loading significantly affected the yield of acrolein. The yield of acrolein increased with an increase in WO_3_ loading; WO_3_/Al_2_O_3_ catalyst with 20 wt% WO_3_ loading showed the highest acrolein yield. On the other hand, excess loading of WO_3_ led to a decrease in the acrolein yield. Notably, γ-Al_2_O_3_ and WO_3_ showed no activity for this reaction, suggesting that WO_3_ loaded on γ-Al_2_O_3_ was the main active site for the dehydration of glycerol to form acrolein.

**Fig. 2:**
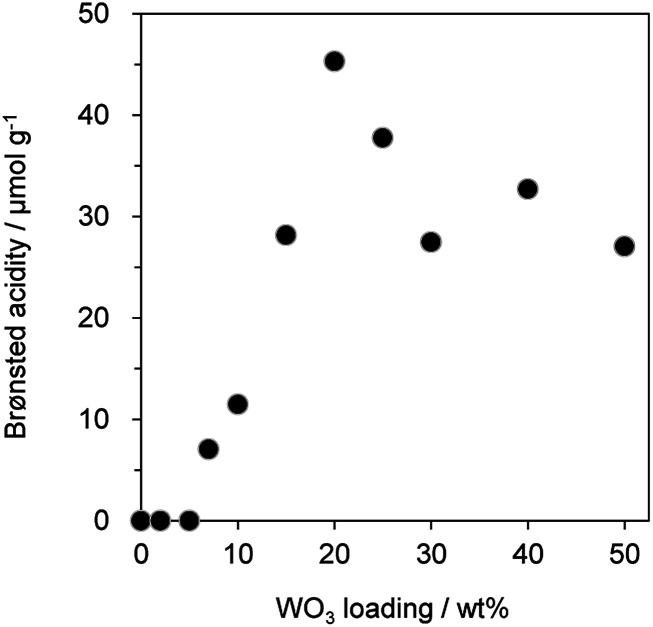
Brønsted acidity of WO_3_/Al_2_O_3_ catalysts with various WO_3_ loadings.

Since previous studies on the relationship between Brønsted acidity and the activity for glycerol dehydration to form acrolein have revealed that Brønsted acid sites were a main active site for acrolein formation,^10,62^ the number of Brønsted acid sites (Brønsted acidity) on WO_3_/Al_2_O_3_ catalysts with various WO_3_ loadings was estimated by pyridine adsorbed FT-IR (Fig. 2 and S2†). Brønsted acidity increased with an increase in WO_3_ loading, and WO_3_/Al_2_O_3_ catalyst with 20 wt% WO_3_ loading exhibited the highest Brønsted acidity. In contrast, the Brønsted acidity decreased gradually for catalysts with loadings above 20 wt%. Acrolein was formed in the highest yield with the catalyst with 20 wt% WO_3_ loading which showed the highest Brønsted acidity. We reported that a two-dimensional WO_3_ monolayer was formed on γ-Al_2_O_3_ when the WO_3_ loading was below 20 wt% and Brønsted acid sites were generated at the boundaries between the WO_3_ domains.^48,49^ The close correlation between the catalytic activity and Brønsted acidity was observed. Some researchers reported that dehydration mechanism of glycerol and the formation of acrolein proceeds over Brønsted acid sites preferentially to over Lewis acid sites.^9,10,16,18,62^ Similarly, the active sites of this reaction should be the Brønsted acid sites at the boundaries between WO_3_ domains on γ-Al_2_O_3_. After protonation of hydroxyl group at the secondary carbon of glycerol by the Brønsted acid sites at the boundaries between WO_3_ domains, the dehydration and keto–enol tautomerism takes place to give 3-hydroxypropionaldehyde. The further dehydration of 3-hydroxypropionaldehyde can easily proceed because of the low stability to produce acrolein.

**Fig. 3:**
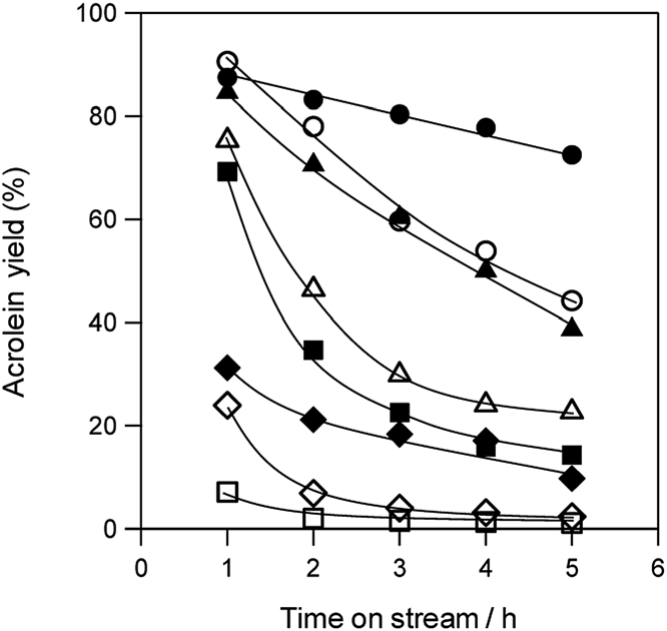
Dehydration of glycerol to acrolein over various acid catalysts. ●: WO_3_/Al_2_O_3_ with 20 wt% WO_3_ loading, ▲: WO_3_/ZrO_2_ with 10 wt% WO_3_ loading, ■: WO_3_/TiO_2_ with 2.5 wt% WO_3_ loading, ◆: Nb_2_O_5_, ○: H-ZSM-5(90), △: H-β(25), □: H-Y(5.5), ◇: H-MOR(20). Conditions: catalyst (100 mg), WHSV by glycerol (4.7 h^−1^), *T* = 588 K.

Fig. 3 shows the durability of various solid acid catalysts in the dehydration of glycerol to acrolein. All the tested catalysts provided acrolein as a main product (Fig. S3†). Although WO_3_/ZrO_2_ with 10 wt% WO_3_,^60^ WO_3_/TiO_2_ with 2.5 wt% WO_3_, H-ZSM-5(90) and H-β(25) showed high acrolein yield at the initial stage of the reaction, the rapid deactivation of these catalysts was observed. H-Y(5.5), H-MOR(20) and Nb_2_O_5_ showed low initial activity. On the other hand, WO_3_/Al_2_O_3_ catalyst with 20 wt% WO_3_ loading showed high catalytic performance regarding both activity and durability.

Coke deposition during dehydration is a well-known cause of catalyst deactivation.^49–54^ Thus, to identify factors that contribute to deactivation, we evaluated the BET surface area of various solid acid catalysts before and after the reaction for 5 h as well as the amount of deposited coke (Table 1). Although coke was deposited on all of the catalyst surfaces, no remarkable relationship was observed between the amount of deposited coke and deactivation rates of catalysts. The BET surface areas of zeolite catalysts were drastically decreased after reaction. On the other hand, the decreases in the surface area of supported WO_3_ catalysts were much smaller than those of zeolite catalysts. Particularly, WO_3_/Al_2_O_3_ catalyst maintained a larger surface area than the other supported WO_3_ catalysts. These results suggest that WO_3_/Al_2_O_3_ catalyst maintained a large surface area during the reaction showed the highest acrolein yield and stability in the dehydration of glycerol.

**Table 1:** Physical properties of various catalysts before and after the reaction for 5 h

Catalyst	Amount of deposited coke^a^/mg g_cat_^−1^	BET surface area/m^2^ g_cat_^−1^	
		Before reaction	After reaction^b^
WO_3_/Al_2_O_3_ (WO_3_: 20 wt%)	131	109	104
WO_3_/ZrO_2_ (WO_3_: 10 wt%)	82	48	69
WO_3_/TiO_2_ (WO_3_: 2.5 wt%)	35	14	18
H-ZSM-5 (90)	138	460	116
H-β (25)	198	660	103
H-Y (5.5)	225	749	25
H-MOR (20)	151	502	14

a Estimated by TG analysis.

b Reaction under flowing N_2_ after 5 h.

**Fig. 4:**
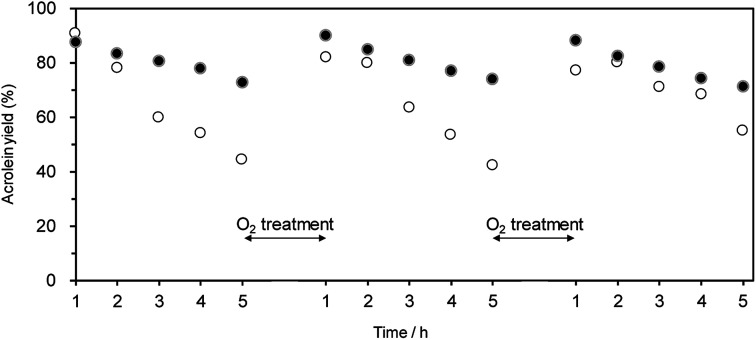
Effect of O_2_ treatment on acrolein yield over (●) WO_3_/Al_2_O_3_ with 20 wt% WO_3_ loading and (○) H-ZSM-5(90) catalysts. Conditions: catalyst (100 mg), WHSV by glycerol (4.7 h^−1^), *T* = 588 K. O_2_ treatment was carried out after 5 h at the temperature at which deposited coke completely decomposed; 823 K (WO_3_/Al_2_O_3_) and 923 K (H-ZSM-5), respectively.

Oxidative treatment is useful for removing the coke deposited at active Brønsted acid sites.^63–65^ Hence, we examined the effect of the oxidative treatment of WO_3_/Al_2_O_3_ and H-ZSM-5(90) after the catalytic run on the regeneration of catalytic performance. Treatment with O_2_ flow (at 823 and 923 K for WO_3_/Al_2_O_3_ and H-ZSM-5(90), respectively) successfully recovered the initial catalytic activity for both WO_3_/Al_2_O_3_ with 20 wt% WO_3_ loading and H-ZSM-5(90) (Fig. 4). This suggests that the coke deposited at active Brønsted acid sites can be removed by oxidative treatment, and WO_3_/Al_2_O_3_ catalyst can be repeatedly used for the dehydration reaction.

**Fig. 5:**
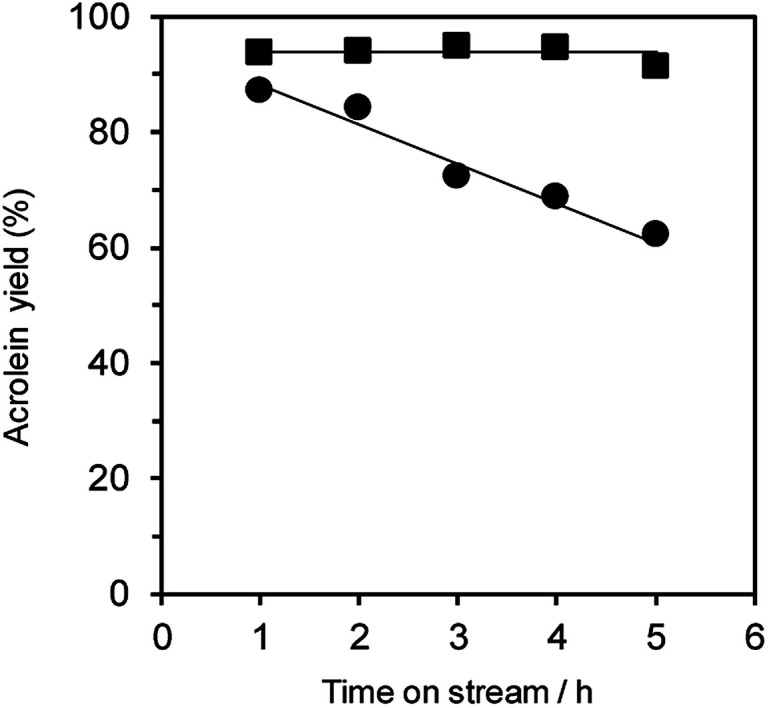
Dehydration of glycerol to acrolein over WO_3_/Al_2_O_3_ catalysts with 20 wt% WO_3_ loading and under flowing N_2_ (circle) and O_2_ (square). Conditions: catalyst (100 mg), WHSV by glycerol (4.7 h^−1^), *T* = 588 K.

Additionally, the dehydration of glycerol over WO_3_/Al_2_O_3_ catalyst with 20 wt% WO_3_ loading (Fig. 5) and H-ZSM-5 (Fig. S4†) under flowing N_2_ and O_2_ gas was investigated. The reaction under O_2_ flow with both WO_3_/Al_2_O_3_ and H-ZSM-5 catalysts showed constant acrolein yields of around 95% for 5 h, indicating that the reaction under an O_2_ atmosphere was effective for protecting the catalysts from deactivation. Note that no excess oxidised products (such as acrylic acid) were obtained in this reaction condition.

**Fig. 6:**
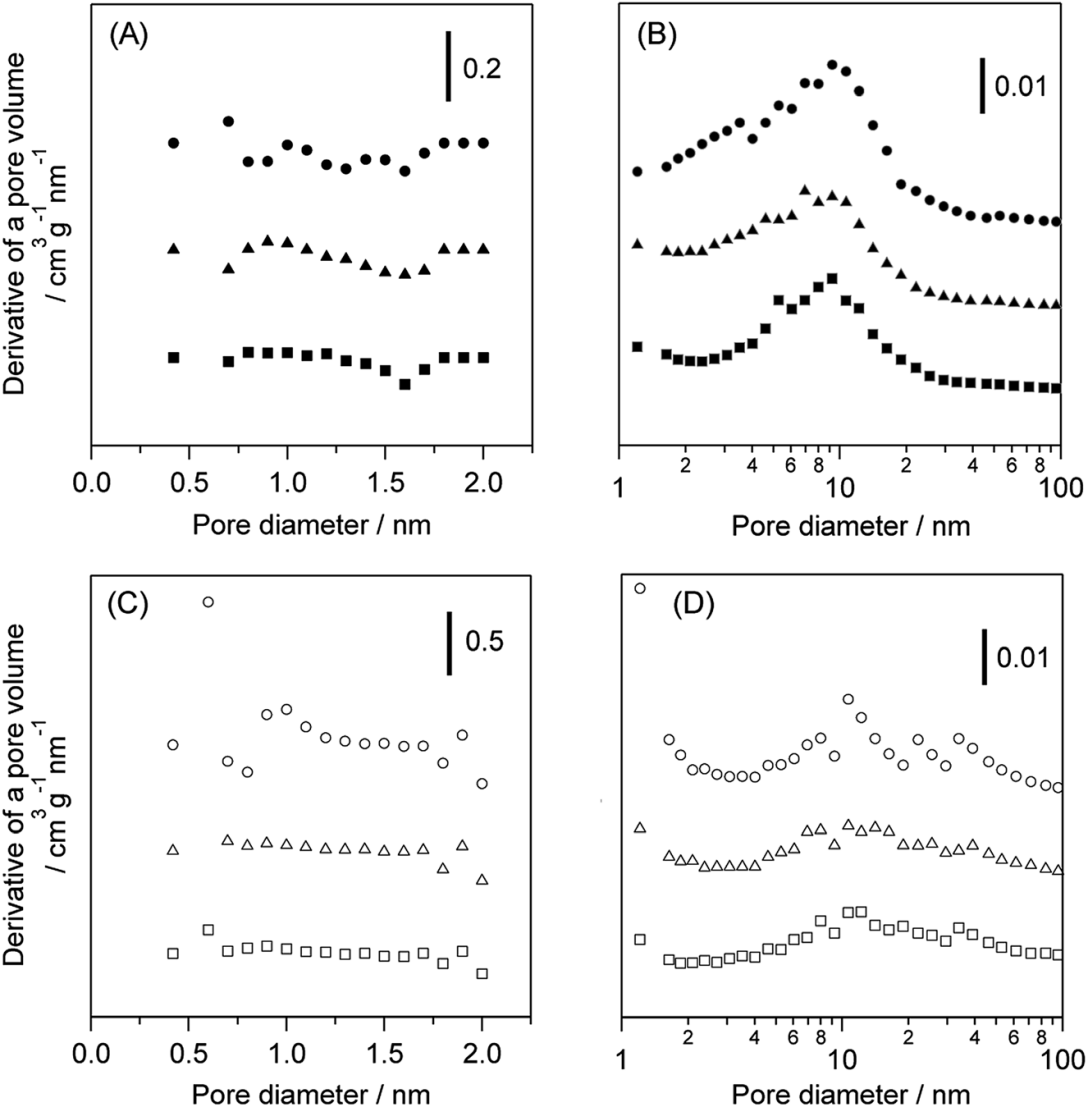
Pore size distribution of the (A) micropore and (B) mesopore regions in WO_3_/Al_2_O_3_ with 20 wt% WO_3_ loading and (C) micropore and (D) mesopore regions in H-ZSM-5(90). Fresh catalyst (circle), after the reaction with N_2_ (triangle) and O_2_ (square) for 5 h.

Table 2 shows the effect of the feed gas on the physical properties of WO_3_/Al_2_O_3_ and H-ZSM-5 catalysts after the catalytic run. The amount of deposited coke in the reaction under O_2_ was greater than that under N_2_ over both catalysts. The acidity of catalysts after the reaction with flowing N_2_ and O_2_ was also estimated by NH_3_-TPD (Fig. S6†). Note that pyridine adsorbed FT-IR measurement could not be used to estimate the acidity because coke deposition on the catalyst caused quite low IR transmittance. No difference in the acidity of the WO_3_/Al_2_O_3_ catalyst between before and after the reaction was observed regardless of the reaction atmosphere. In contrast, the area of NH_3_ desorption peak at 673 K was drastically decreased, indicating that the number of strong acid sites of H-ZSM-5 was decreased by the reaction. (Fig. S6(B)).† The micropore and mesopore distributions of WO_3_/Al_2_O_3_ and H-ZSM-5 catalysts are shown in Fig. 6. No micropores were observed in the WO_3_/Al_2_O_3_ catalyst. Furthermore, no significant change in pore size distribution after the reaction under both N_2_ and O_2_ was observed. On the other hand, H-ZSM-5 showed large number of micropores and mesopores. The catalytic runs drastically decreased the number of micropores in H-ZSM-5. These results suggest that coke deposition at the entrance of the H-ZSM-5 pore reduced the ability of glycerol to access the acid sites inside the micropores. Based on these results, we can conclude that the high activity and durability of WO_3_/Al_2_O_3_ are responsible for the fact that the mesoporous nature of WO_3_/Al_2_O_3_ was not prevented by coke formation at the entrance of pores and Brønsted acid sites.

**Table 2:** Physical properties of WO_3_/Al_2_O_3_ with 20 wt% WO_3_ loading and H-ZSM-5(90) catalysts after reaction under flowing N_2_ and O_2_

Catalyst	Carrier gas	Amount of deposited coke^a^/mg g_cat_^−1^	BET surface area/m^2^ g_cat_^−1^		Acidity^b^/μmol g_cat_^−1^	
			Before reaction	After reaction^c^	Before reaction	After reaction^c^
WO_3_/Al_2_O_3_ (WO_3_: 20 wt%)	N_2_	131	109	104	296	104
	O_2_	221		69		297
H-ZSM-5 (90)	N_2_	138	460	116	158	48
	O_2_	170		131		30

a Estimated by TG analysis.

b Estimated by NH_3_-TPD.

c Reaction after 5 h.

**Fig. 7:**
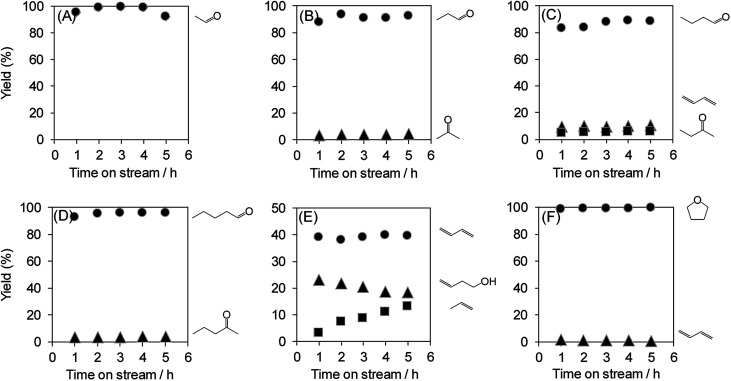
Dehydration of various polyols over WO_3_/Al_2_O_3_ catalysts with 20 wt% WO_3_ loading. Conditions: catalyst (100 mg), WHSV by substrate (4.7 h^−1^), *T* = 588 K. (A) Ethylene glycol, (B) 1,2-propanediol, (C) 1,2-butanediol, (D) 1,2-pentanediol, (E) 1,3-butanediol and (F) 1,4-butanediol.

The dehydration of polyols other than glycerol can also provide useful chemicals. Therefore, the dehydration of various polyols over WO_3_/Al_2_O_3_ catalyst with 20 wt% WO_3_ loading was investigated (Fig. 7). The reactions of polyols gave the corresponding aldehydes, dienes, ketones and ethers. In particular, the dehydration of polyols with hydroxyl groups at the 1,2-position such as glycerol, ethylene glycol, 1,2-propanediol, 1,2-butanediol and 1,2-pentanediol afforded the aldehydes (*e.g.* acrolein, acetaldehyde, propionaldehyde, butyraldehyde and valeraldehyde) as main products. On the other hand, the reaction of 1,3-butanediol mainly produced 1,3-butadiene through double dehydration. In contrast, the dehydration of 1,4-butanediol gave tetrahydrofuran as a sole product. These results indicate that WO_3_/Al_2_O_3_ catalyst with 20 wt% WO_3_ loading shows high activity and durability for the dehydration of various polyols.

## Conclusions

Gas-phase dehydration of polyols over WO_3_/Al_2_O_3_ catalysts was investigated. WO_3_ loading on γ-Al_2_O_3_ strongly affected the activity for the dehydration of glycerol to acrolein, and WO_3_/Al_2_O_3_ catalyst with 20 wt% WO_3_ loading showed the highest acrolein yield. Brønsted acid site on the WO_3_/Al_2_O_3_ surface catalysed the formation of acrolein with high selectivity. WO_3_/Al_2_O_3_ catalyst with 20 wt% WO_3_ loading showed a higher product yield than the other supported WO_3_ catalysts and zeolites. H-ZSM-5 has both micro- and mesopores and coke deposits at the entrances of those pores. On the other hand, WO_3_/Al_2_O_3_ catalysts have no micropores, suggesting that glycerol can access surface Brønsted acid sites at the inside of the mesopores of WO_3_/Al_2_O_3_ and acrolein is continuously formed with high durability during the reaction. No decrease in acrolein yield was observed under O_2_ flow during dehydration over WO_3_/Al_2_O_3_ catalyst. On the other hand, the amount of deposited coke under O_2_ flow was greater than that under N_2_, implying that coke deposited at the catalyst surface was removed by O_2_. Furthermore, WO_3_/Al_2_O_3_ catalyst with 20 wt% WO_3_ loading showed high activity and durability for the dehydration of various polyols.

## Conflicts of interest

There are no conflicts to declare.

## Supplementary Material

RA-010-D0RA08340B-s001
