# Supplementary material for: Highly active and durable WO3/Al2O3 catalysts for gas-phase dehydration of polyols
Source: RSC Adv. 2020 Oct 12;10(61):37538–44. doi: 10.1039/d0ra08340b (PMC9057158; doi:10.1039/d0ra08340b)
Supplement: RA-010-D0RA08340B-s001 [file RA-010-D0RA08340B-s001.pdf]

Electronic Supplementary Material (ESI) for RSC Advances.
This journal is © The Royal Society of Chemistry 2020

Supporting Information

Highly Active and Durable WO_3/Al_2O_3 Catalysts for Gas-phase Dehydration of Polyols

Takeshi Aihara[a], Katsuya Asazuma[a], Hiroki Miura[a,b,d], and Tetsuya Shishido[a,b,c,d*]

[a] Department of Applied Chemistry for Environment, Graduate School of Urban Environmental Sciences, Tokyo Metropolitan University, 1-1 Minami-Osawa, Hachioji, Tokyo 192-0397, Japan

[b] Research Center for Hydrogen Energy-based Society, Tokyo Metropolitan University, 1-1 Minami-Osawa, Hachioji, Tokyo 192-0397, Japan

[c] Research Center for Gold Chemistry, Tokyo Metropolitan University, 1-1 Minami-Osawa, Hachioji, Tokyo 192-0397, Japan

[d] Elements Strategy Initiative for Catalysts & Batteries, Kyoto University, Katsura, Nishikyo-ku, Kyoto 615-8520, Japan

[*] Corresponding author: Tel: +81-42-677-2850 Fax: +81-42-677-2850 (T. Shishido)

E-mail address: shishido-tetsuya@tmu.ac.jp (T. Shishido)

Analytical data

Contents:

1. IR spectra of pyridine adsorbed on acid sites of WO_3/Al_2O_3 catalysts.

2. Conversion, Yield and Selectivty.

3. Selectivity of acrolein produced from glycerol over various solid acid catalysts.

4. TG analysis of WO_3 loaded catalysts and zeolites after catalitic run under N_2.

5. Dehydration of glycerol over H-ZSM-5 under N_2 and O_2.

6. TG analysis of WO_3/Al_2O_3 and H-ZSM-5 after catalytic cycle under O_2.

7. NH_3-TPD profiles of WO_3/Al_2O_3 and H-ZSM-5 before and after the reaction under N_2 and O_2.

8. Previous studies on gas-phase dehydration of polyols.

References

1. Difference spectra of pyridine adsorbed on acid sites of WO_3/Al_2O_3 catalysts

The acidity of WO_3/Al_2O_3 catalysts with variousWO_3 loadings was investigated by adsorbed pyridine on catalysts surface. The bands at 1540 and 1450 cm^{-1} were attributed to pyridinium ion on Brønsted acid site and pyridine coordinated to Lewis acid site, respectively. The amount of Brønsted and Lewis acidity were estimated from the area of bands at 1540 and 1450 cm^{-1} and their integrated molar extinction coefficients; 1.67 and 2.22 cm μmol^{-1}, respectively.[1]

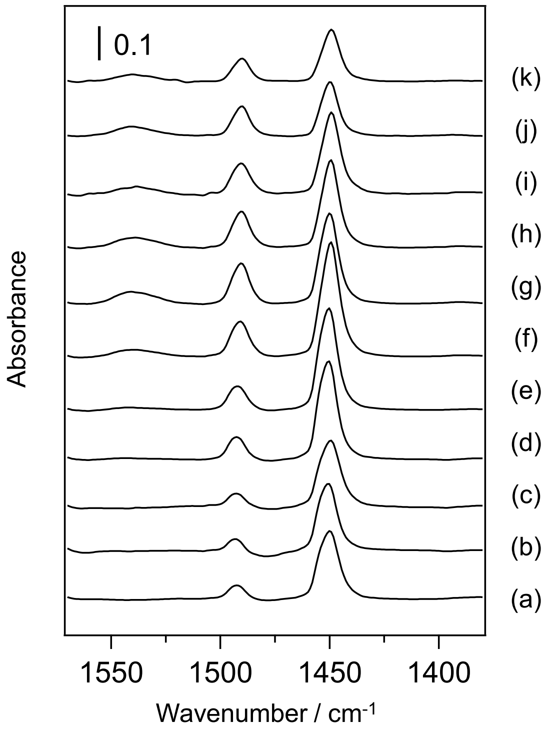

Figure S1. Difference spectra of pyridine adsorbed on WO_3/Al_2O_3 catalysts with various WO_3 loadings. a) 0, b) 2, c) 5, d)7, e) 10, f) 15, g) 20, h) 25, i) 30, j) 40 and k) 50 wt% WO_3 loading.

2. Conversion, Yield and Selectivity

$$Conversion\ (\%) = \frac{sum\ of\ moles\ of\ all\ products}{sum\ of\ moles\ of\ reactant\ and\ all\ products} \times 100$$

$$Yield\ (\%) = \frac{moles\ of\ specific\ product}{sum\ of\ moles\ of\ reactant\ and\ all\ products} \times 100$$

$$Selectivity\ (\%) = \frac{moles\ of\ carbon\ in\ specific\ product}{sum\ of\ moles\ of\ carbon\ in\ reactant\ and\ all\ products} \times 100$$

3. Selectivity of acrolein produced from glycerol over various solid acid catalysts.

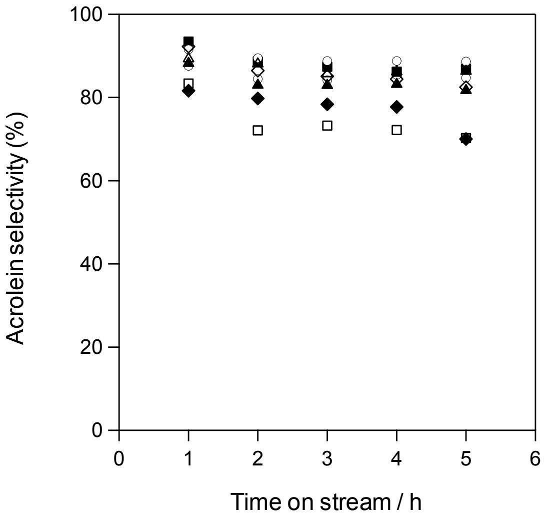

Figure S2. Acrolein selectivity of glycerol dehydration over various solid acid catalysts. ●: WO_3/Al_2O_3 with 20 wt% WO_3 loading, ▲: WO_3/ZrO_2 with 10 wt % WO_3 loading, ■: WO_3/TiO_2 with 2.5 WO_3 wt% loading, ◆: Nb_2O_5, ○: H-ZSM-5(90), △: H-β(25), □: H-Y(5.5), ◇: H-MOR(20). Conditions: Catalyst (100 mg), WHSV by glycerol (4.7 h^{-1}), T= 588 K.

4. TG analysis of WO_3 loaded catalysts and zeolites.

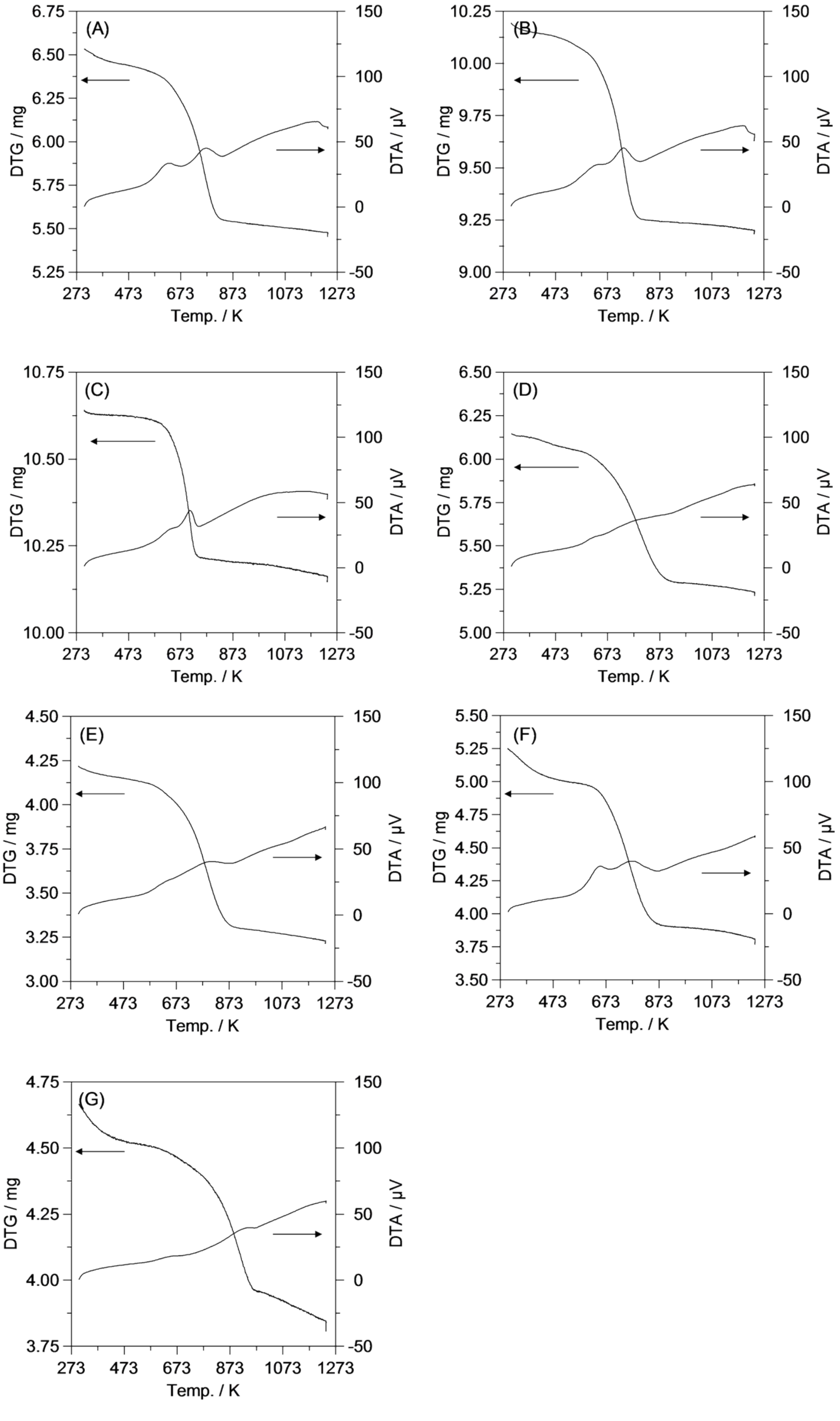

Figure S3. TG analysis of various catalysts after using reaction for 5 h. (A) WO_3/Al_2O_3 with 20 wt% WO_3 loading, (B) WO_3/ZrO_2 with 10 wt% WO_3 loading, (C) WO_3/TiO_2 with 2.5 wt% WO_3 loading, (D) H-ZSM-5(90), (E) H-β(25), (F) H-Y(5.5) and (G) H-MOR(20).

5. Dehydration of glycerol over H-ZSM-5 under N_2 and O_2.

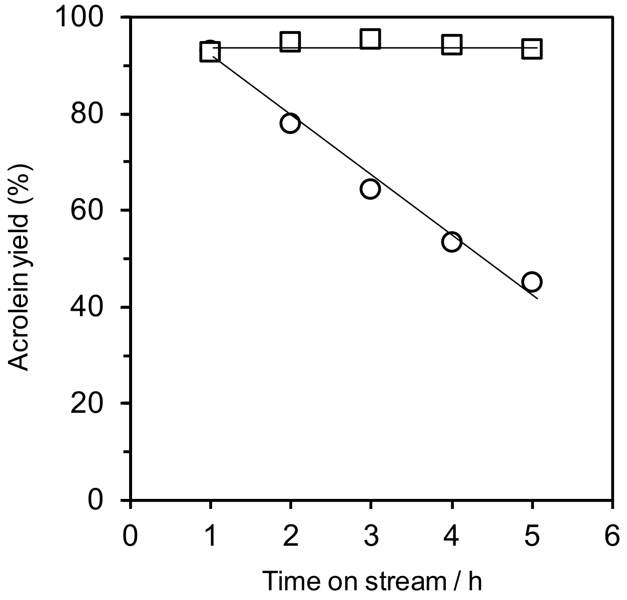

Figure S4. Dehydration of glycerol to acrolein over Dehydration of glycerol to acrolein over H-ZSM-5(90) under flowing N_2 (circle) and O_2 (square). Conditions: Catalyst (100 mg), WHSV by glycerol (4.7 h^{-1}), T= 588 K.

6. TG analysis of WO_3/Al_2O_3 and H-ZSM-5 after catalytic cycle under O_2.

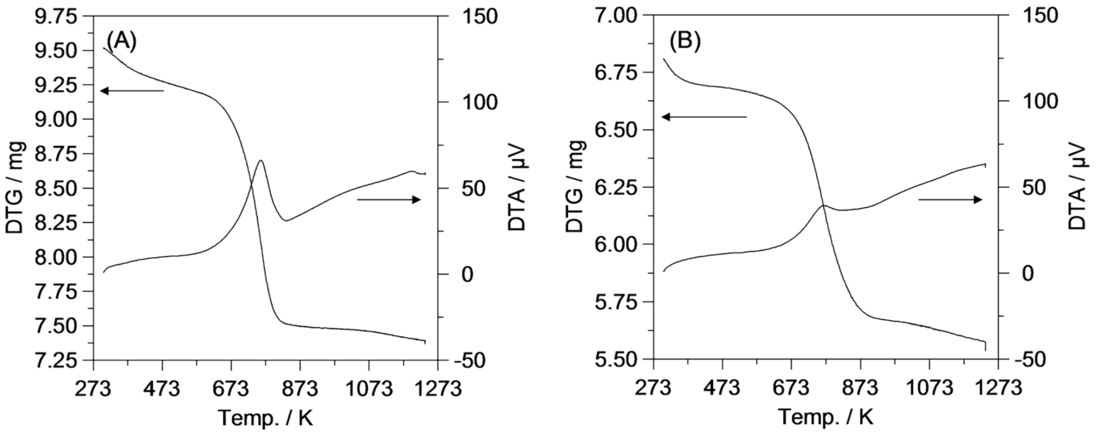

Figure S5. TG analysis of (A) WO_3/Al_2O_3 with 20 wt% WO_3 loading and (B) H-ZSM-5(90) catalysts after reaction for 5 h under flowing O_2.

7. NH_3-TPD profiles of WO_3/Al_2O_3 and H-ZSM-5 before and after the reaction under N_2 and O_2.

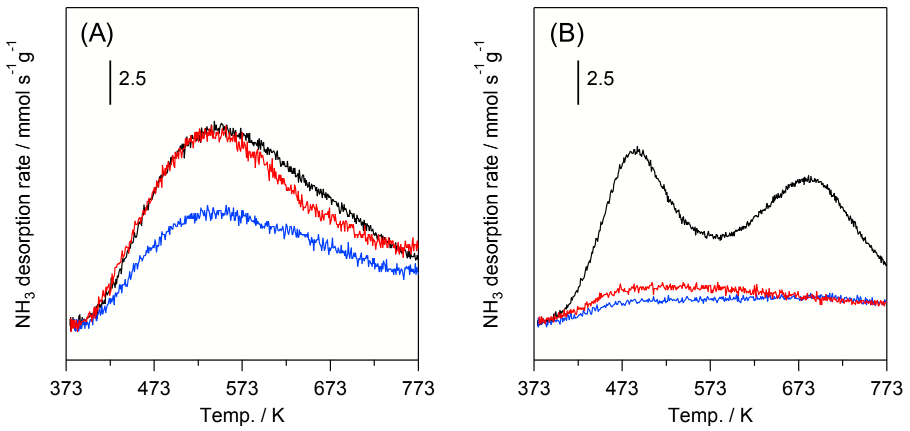

Figure S6. NH_3-TPD profiles of (A) 20 wt% WO_3/Al_2O_3 and (B) H-ZSM-5(90). —: flesh, —: after the reaction under N_2 flow and —: after reaction under O_2 flow.

8. Previous studies on gas-phase dehydration of polyols

Table S1. Comparison of reaction results on gas-phase dehydration of polyols.

Catalyst	Catalyst weight / mg	Substrate	Fed rate / mL h^{-1}	Carrier	Temp. / K	Main product	Yield (%) at 1 h	Yield (%) at 5 h	Yield ratio (5 h / 1 h)	Reference
20 wt% WO_3/Al_2O_3	100	Glycerol	0.37	N_2	588	Acrolein	85	72	0.84	This Work
20 wt% WO_3/Al_2O_3	100	Glycerol	0.37	O_2	588	Acrolein	93	90	0.97	This Work
H-ZSM-5 (SiO_2/Al_2O_3=90)	100	Glycerol	0.37	N_2	588	Acrolein	90	45	0.50	This Work
Nb_2O_5 (Cal. 673 K)	570	Glycerol	50.4	N_2	588	Acrolein	37	47	1.27	S2
Nb_2O_5 (Cal. 973 K)	840	Glycerol	50.4	N_2	588	Acrolein	10	11	1.10	S2
H-ZSM-5 (SiO_2/Al_2O_3=90)	380	Glycerol	50.4	N_2	588	Acrolein	32 (1–2 h)	14 (9–10 h)	0.44	S3
15 wt% WO_3/Al_2O_3	310	Glycerol	0.37	N_2	588	Acrolein	46	68	1.48	S4
15 wt% WO_3/ZrO_3	630	Glycerol	0.37	N_2	588	Acrolein	53	63	1.19	S4
23 wt% WO_3/SiO_2	330	Glycerol	0.37	N_2	588	Acrolein	47	30	0.64	S4
MFI zeolite (SiO_2/Al_2O_3=14)	150	Glycerol	1.33	N_2	578	Acrolein	20	6	0.30	S5
MFI zeolite (SiO_2/Ga_2O_3=23)	150	Glycerol	1.33	N_2	578	Acrolein	13	6	0.46	S5
BPO_4	220	1,2-Propanediol	0.53	H_2	493	Propanal	90	75	0.83	S6
30 wt% $H_4SiW_{12}O_{40}$ /SiO_2	300	1,2-Propanediol	1.70	N_2	473	Propanal	67 (Average)		-	S7
SiO_2-SI_2O_3	300	1,2-Propanediol	1.70	N_2	523	Propanal	29 (Average)		-	S7
ZrO_2	300	1,2-Propanediol	1.70	N_2	673	Propanal	21 (Average)		-	S7
TiO_2	300	1,2-Propanediol	1.70	N_2	623	Acetone	22 (Average)		-	S7
Amberlyst-15	300	1,2-Propanediol	1.70	N_2	473	Dioxolane	49 (Average)		-	S7
Al_2O_3	300	1,2-Propanediol	1.70	N_2	573	Dioxolane	28 (Average)		-	S7
CeO_2	150	Glycerol	2.01	N_2	698	1-hydroxy-2-propanone	28 (Average)		-	S8
CeO_2	150	1,2-Butanediol	2.01	N_2	698	Butanal	8 (Average)		-	S8
CeO_2	150	1,3-Butanediol	2.01	N_2	698	3-buten-2-ol	46 (Average)		-	S8
CeO_2	150	1,4-Butanediol	2.01	N_2	698	3-buten-1-ol	40 (Average)		-	S8
CeO_2	150	2.3-Butanediol	2.01	N_2	698	Butanone	33 (Average)		-	S8
CeO_2	150	1,2-Propanediol	2.01	N_2	698	1-Propanol	9 (Average)		-	S8
CeO_2	150	1,3-Propanediol	2.01	N_2	698	2-Propen-1-ol	42 (Average)		-	S8
CeO_2	150	2,4-Pentanediol	2.01	N_2	698	*trans*-3-penten-2-ol	59 (Average)		-	S8
In_2O_3	500	1,3-propanediol	2.67	N_2	648	2-propen-1-ol	72	22	0.31	S9
In_2O_3	500	1,4-butanediol	2.67	N_2	648	3-buten-1-ol	56	56	1.00	S9

References

S1. C. A. Emeis, *J. Catal.*, 1993, **141**, 347–354.

S2. S. Chai, H. Wang, Y. Liang and B. Xu, *J. Catal.*, 2007, **250**, 342–349.

S3. S.-H. Chai, H.-P. Wang, Y. Liang and B.-Q. Xu, *Green Chem.*, 2007, **9**, 1130–1136.

S4. S.-H. Chai, B. Yan, L.-Z. Tao, Y. Liang and B.-Q. Xu, *Catal. Today*, 2014, **234**, 215–222.

S5. L. H. Vieira, K. T. G. Carvalho, E. A. Urquieta-González, S. H. Pulcinelli, C. V. Santilli and L. Martins, *J. Mol. Catal. Chem.*, 2016, **422**, 148–157.

S6. R. Otomo, C. Yamaguchi, D. Iwaisako, S. Oyamada and Y. Kamiya, *ACS Sustain. Chem. Eng.*, 2019, **7**, 3027–3033.

S7. K. Mori, Y. Yamada and S. Sato, *Appl. Catal. Gen.*, 2009, **366**, 304–308.

S8. S. Sato, R. Takahashi, T. Sodesawa and N. Honda, *J. Mol. Catal. Chem.*, 2004, **221**, 177–183.

S9. M. Segawa, S. Sato, M. Kobune, T. Sodesawa, T. Kojima, S. Nishiyama and N. Ishizawa, *J. Mol. Catal. Chem.*, 2009, **310**, 166–173.
